# Cooperation and Cheating through a Secreted Aminopeptidase in the Pseudomonas aeruginosa RpoS Response

**DOI:** 10.1128/mBio.03090-19

**Published:** 2020-03-17

**Authors:** Tanner Robinson, Parker Smith, Erin R. Alberts, Mariana Colussi-Pelaez, Martin Schuster

**Affiliations:** a Department of Microbiology, Oregon State University, Corvallis, Oregon, USA; National Institute of Child Health and Human Development (NICHD)

**Keywords:** stress response, RpoS, quorum sensing, proteolysis, *Pseudomonas aeruginosa*, cooperation, cheating, experimental evolution

## Abstract

The global stress response controlled by the alternative sigma factor RpoS protects enteric bacteria from a variety of environmental stressors. The role of RpoS in other, nonenteric bacteria, such as the opportunistic pathogen Pseudomonas aeruginosa, is less well understood. Here, we employed experimental social evolution to reveal that cooperative behavior via secreted public goods is an important function in the RpoS response of P. aeruginosa. Using whole-genome sequencing, we identified *rpoS* loss-of-function mutants among isolates evolved in a protein growth medium that requires extracellular proteolysis. We found that *rpoS* mutants comprise up to 25% of the evolved population and that they behave as social cheaters, with low fitness in isolation but high fitness in mixed culture with the cooperating wild type. We conclude that *rpoS* mutants cheat because they exploit an RpoS-controlled public good produced by the wild type, the secreted aminopeptidase PaAP, and because they do not carry the metabolic costs of expressing PaAP and many other gene products in the large RpoS regulon. Our results suggest that PaAP is an integral part of a proteolytic sequence in P. aeruginosa that permits the utilization of protein as a nutrient source. Our work broadens the scope of stress response functions in bacteria.

## Abstract (precis)

Bacterial stress responses are generally considered protective measures taken by individual cells. Enabled by an experimental evolution approach, we describe a contrasting property, collective nutrient acquisition, in the RpoS-dependent stress response of the opportunistic human pathogen P. aeruginosa. Specifically, we identify the secreted P. aeruginosa aminopeptidase (PaAP) as an essential RpoS-controlled function in extracellular proteolysis. As a secreted “public good,” PaAP permits cheating by *rpoS* mutants that save the metabolic costs of expressing RpoS-controlled genes dispensable under the given growth conditions. Proteolytic enzymes are important virulence factors in P. aeruginosa pathogenesis and constitute a potential target for antimicrobial therapy. More broadly, our work contributes to recent findings in higher organisms that stress affects not only individual fitness and competitiveness but also cooperative behavior.

## INTRODUCTION

Stress responses allow bacteria to adapt to adverse environmental conditions (1). Based on a few well-understood model systems, the literature in this area has focused on processes that help cells cope with stress, primarily protection, dormancy, and repair (1). Bacteria may employ responses targeted to certain specific stresses, or they may globally alter their metabolism and gene expression to resist many different types of stresses. The general stress response dependent on the alternative sigma factor RpoS is one such global response (2). RpoS is conserved among proteobacteria, although the specific functions controlled by RpoS differ (3). In Escherichia coli, the RpoS response helps cells to cope with nutrient exhaustion in stationary phase but also provides cross-protection in response to other stresses such as reactive oxygen species, extreme temperature, and extreme pH (2, 4). The relevant ecological trigger of RpoS induction may be interference competition from other microbes, a phenomenon termed “competition sensing” (5). While RpoS provides vitally important stress protection, its presence is unfavorable under certain growth conditions. RpoS mutants have an advantage under conditions of moderate nutrient limitation (6) and extreme starvation in late stationary phase (termed the “GASP phenotype”) (7). RpoS mutations initially reported in natural populations of enteric bacteria (8, 9) may, however, be largely an artifact of subsequent laboratory storage (10).

Beyond E. coli and other enteric bacteria, the role of RpoS is less well understood. In the opportunistic pathogen Pseudomonas aeruginosa, RpoS controls over 700 genes, broadly distributed over many functional categories (11). Even though RpoS is induced in stationary phase, its role in general stress resistance is less pronounced than in E. coli (12, 13). RpoS disproportionately affects extracellular secretions, albeit without a discernible pattern, inducing some (e.g., the exopolysaccharide alginate and the protein toxin exotoxin A) and repressing others (e.g., the secondary metabolites cyanide and pyocyanin) (13, 14).

Secreted products can be considered “public goods” that mediate widespread cooperative behaviors in microbes (15). P. aeruginosa secretes a range of public goods, many of which are controlled by a cell-cell signaling mechanism termed quorum sensing (QS) (16). These include extracellular proteases that hydrolyze peptide bonds in proteins and peptides. As such, proteases represent an important virulence factor that destroys host tissue and interferes with immune function (17). They permit growth on protein as the sole carbon (C) source *in vitro* and *in vivo* (18). Under growth conditions that require extracellular proteolysis, nonproducing mutants evolve that take advantage of the proteases produced by others in the population (19, 20), highlighting the general phenomenon of “cheating” in microbial populations (21). The protease-negative mutants are deficient in the major QS regulator LasR, permitting continued quorum signal synthesis without signal reception (19, 20). In addition to cheating, cooperative proteolytic growth selects for nonsocial adaptations (22, 23). A loss-of-function mutation in the transcriptional regulator PsdR derepresses an amino acid and dipeptide uptake operon, increasing the processing of the breakdown products of extracellular proteolysis (22, 24).

In the present study, we comprehensively identified adaptations during cooperative growth of P. aeruginosa by sequencing the genomes of 30 evolved isolates from our previous studies (20, 23). We focused on the characterization of widespread mutations in *rpoS*. Surprisingly, these loss-of-function mutations confer a cheater phenotype. They prevent growth in isolation but provide a substantial fitness advantage in coculture with *rpoS*-proficient cells. Among the more than 700 genes controlled by P. aeruginosa RpoS, we identified a single gene, *pepB*, as essential for cooperative, proteolytic growth. This gene encodes the secreted P. aeruginosa aminopeptidase PaAP. PaAP has been characterized biochemically as a leucine aminopeptidase that preferentially catalyzes the hydrolysis of leucine from the N terminus of peptides, but its metabolic role is largely unknown (25–27). Our data assign a cooperative function to the RpoS regulon in P. aeruginosa and, more generally, highlight a role for stress responses beyond cellular protection, dormancy, and repair.

## RESULTS

### RpoS mutants in a social evolution experiment.

In our previous *in vitro* evolution experiments, the P. aeruginosa PAO1 wild-type (WT) strain was grown in casein minimal medium for 20 days, with subculturing performed every day (20, 23). The milk protein casein was provided as the sole C source, requiring QS-dependent extracellular proteolysis for population growth. Here, we sequenced the genomes of 30 evolved isolates from the final day of subculturing, including all nine isolates available from the earlier study by Sandoz et al. and 21 isolates from the more recent study by Wilder et al. (two replicate lines each [20, 23]). *In vitro* evolution experiments in both studies were conducted in the same laboratory under identical culture conditions. We grouped the isolates into three classes based on two QS-dependent phenotypes, skim milk proteolysis and growth on adenosine as the sole C source (Table 1). Adenosine utilization offers an additional, easily scorable phenotype that directly depends on a functional LasR (28). Our classification confirmed previous results (20, 23). Class I isolates are protease and adenosine positive, class II isolates are protease positive but adenosine negative, and class III isolates are both protease and adenosine negative. Representatives of these three classes were observed in all four replicate experiments. We had previously interpreted class I isolates as QS-proficient “cooperators” with intact LasR, class II isolates as moderately QS-deficient “hybrids” with partially functional LasR, and class III isolates as fully QS-deficient “cheaters” with nonfunctional LasR (20, 22, 23). We report below that the interpretation of class I isolates as cooperators requires revision.

**TABLE 1:** Characteristics of sequenced isolates

Isolate^*a*^	Replicate^*b*^	Phenotype^*c*^			Gene name	Mutation (position)^*d*^	
		Skim milk	Adenosine	NAG		Gene	Protein
Class I							
TR02	1	+	+	*+*	*lasR*	C→T (683)	A228V
					*rpoS*	G→A (922)	R308T
					*psdR*	Δ512–529	Δ172–177
TR03	2	+	+	*+*	*psdR*	Δ512–529	Δ172–177
TR04	2	+	+	*+*	*psdR*	A→C (432)	Y144S
TR05	2	+	+	*+*	*psdR*	A→C (432)	Y144S
TR11	3	+	+	*−*	*rpoS*	G→T (883)	E295*
					*psdR*	T→C (166)	S56P
TR12	3	+	+	*−*	*rpoS*	G→T (883)	E295*
					*psdR*	T→C (166)	S56P
TR13	3	+	+	*−*	*rpoS*	C→T (629)	P210L
					*psdR*	C→A (109)	Q37K
TR14	3	+	+	*+*	*psdR*	G→A (397)	G133R
TR15	3	+	+	*+*	*psdR*	G→A (397)	G133R
TR22	4	+	+	*−*	*rpoS*	Δ58	Frameshift (20)
					*psdR*	T→C (100)	F34L
TR23	4	+	+	*+*	*psdR*	Δ144–147	Frameshift (49)
TR24	4	+	+	*+*	*pslB*	+ CCCGGG (378)	+PG (127)
					*psdR*	Δ144–147	Frameshift (49)
TR25	4	+	+	*−*	*rpoS*	+AG (593)	Frameshift (198)
					*psdR*	A→C (432)	Y144S
TR26	4	+	+	*+*	*psdR*	+A (375)	Frameshift (126)

Class II							
TR06	1	−	−	*+*	*fleQ*	C→T (952)	R318C
					*lasR*	G→A (541)	E181K
					*psdR*	Δ512–529	Δ172–177
TR07	2	−	−	*+*	*fliR*	Δ51	Frameshift (17)
					PA3330	A→G (568)	S190G
					*psdR*	Δ512–529	Δ172–177
TR08	2	−	−	+	Upstream *lasR*	+C (−15)	None
					*psdR*	Δ512–529	Δ172–177
TR16	3	−	−	*−*	*lasR*	Δ645–647	ΔI215
					*rpoS*	C→T (852)	G284S
					*psdR*	C→A (411)	Y137*
TR17	3	−	−	*+*	*lasR*	C→A (475)	A158E
					*psdR*	G→A (397)	G133R
TR27	4	−	−	*+*	*flgI*	C→T (313)	Q105*
					*lasR*	Δ330–342	Δ110–113

Class III							
TR09	1	+	−	+	*lasR*	C→T (683)	A228V
					*psdR*	Δ512–529	Δ172–177
TR10	2	+	−	*+*	*lasR*	C→T (344)	T115I
					Intergenic	G→A (3,559,339^*e*^)	None
					*psdR*	Δ512–529	Δ172–177
TR18	3	+	−	*+*	*lasR*	A→G (634)	M212V
					*psdR*	#	#
TR19	3	+	−	*+*	PA1194	G→A (414)	V138H
					*lasR*	A→G (634)	M212V
					*psdR*	#	#
TR20	3	+	−	*+*	*lasR*	A→G (634)	M212V
					*psdR*	#	#
TR21	3	+	−	*+*	*lasR*	A→G (634)	M212V
					*psdR*	#	#
TR28	4	+	−	−	PA2228	G→T (982)	D328Y
					*ctpH*	G→T (1300)	A434S
					*rpoS*	G→A (3)	M1I
					*psdR*	+A (375)	Frameshift (126)
TR29	4	+	−	*+*	*mutL*	Δ1179–1191	Δ393–397
					*psdR*	+A (375)	Frameshift (126)
TR30	4	+	−	*+*	*lasR*	A→T (605)	N202I
					PA4037	A→C (915)	E305D
					*psdR*	Δ146–149	Frameshift (49)
TR31	4	+	−	*+*	*lasR*	A→T (605)	N202I
					PA2434	A→G (188)	Q63P
					*psdR*	Δ146–149	Frameshift (49)

a Isolates are classified according to phenotype. Class I, skim milk proteolysis and adenosine positive; class II, skim milk proteolysis and adenosine negative; class III, skim milk proteolysis positive and adenosine negative. TR01, the sequenced PAO1 WT parent, is not listed here.

b Numbers indicate replicates from *in vitro* evolution experiments previously reported as follows: 1 and 2, reference 20; 3 and 4, reference 23.

c +, proteolysis positive on skim milk, growth positive on adenosine or NAG; −, no proteolysis on skim milk, no or marginal growth on adenosine or NAG.

d Nucleotide substitution (→), insertion (+), or deletion (Δ) at the indicated position relative to the translational start site of the corresponding gene in the P. aeruginosa PAO1 genome, as well as the corresponding amino acid change in the resulting protein. *, stop codon; #, predicted mutation not identified due to contig gap in genome alignment.

e Chromosomal coordinates are given for this intergenic locus.

Sequencing was performed on an Illumina HiSeq 3000 platform with 150-bp paired-end reads. After low-quality reads were trimmed and sequences were aligned to the parent PAO1 strain as a reference, mutations were called and assessed (Table 1; see also Fig. S1 in the supplemental material). Sequencing revealed *psdR* mutations in all but five isolates. Upon closer examination, we found that a contig gap in the assembled genome fell in the middle of the *psdR* gene in four of the five cases. Due to this and the discovery of mutations at this locus in all of the other isolates, we believe that each isolate with a contig gap does indeed contain a mutation in this gene. This interpretation is consistent with our previous work, which characterized widespread loss of PsdR as an important adaptation to the proteolytic growth environment (22). In the class III genomes, we found a mutation in the *lasR* coding region in five of six isolates. In the remaining isolate, there was an insertion in the −15 position from the *lasR* translation start site that presumably disrupts translation by hampering ribosome binding. Class II genomes had mutations in *lasR* and *psdR*, in addition to other loci that were not consistent throughout the group. The abundance of *lasR* mutations in these two classes and in all four replicate experiments also confirms our previous work (20, 22, 23). Importantly, in the class I genomes we found a mutation in *rpoS* in 6 of 14 isolates (43%) in three of four replicate experiments. An *rpoS* mutation was identified in only one class II isolate and one class III isolate. This was the only novel mutation consistent within a group, making it a compelling candidate for further study.

10.1128/mBio.03090-19.1 FIG S1 Isolate mutation map. The chromosomal positions of all identified mutations in each sequenced P. aeruginosa isolate are shown. The coordinates of the PAO1 reference genome are indicated along the top, with blue bars placed every 1,000,000 bp. Isolates are divided by class designations as indicated on the left side. Individual mutations (yellow ovals) are marked in line with each isolate at the respective genome positions. The positions of the genes with the major mutations, *lasR*, *rpoS*, and *psdR*, are indicated along the bottom. A number sign (#) indicates a contig gap in the *psdR* gene with a presumed mutation. See Table 1 in the main text for a detailed description of mutations. Download FIG S1, PDF file, 0.03 MB. Copyright © 2020 Robinson et al. 2020 Robinson et al. This content is distributed under the terms of the Creative Commons Attribution 4.0 International license.

**FIG 1:**
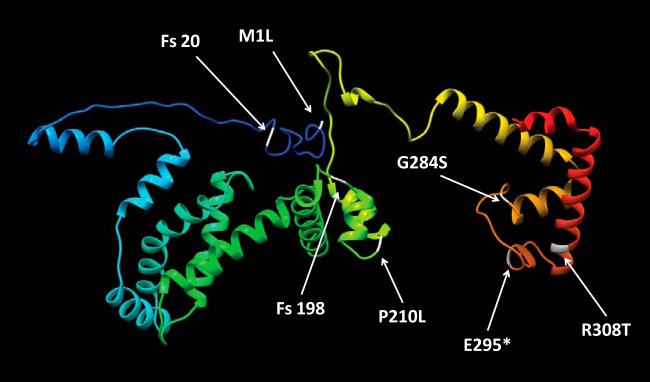
RpoS protein structure and identified mutations. The ribbon diagram shows a model of RpoS from P. aeruginosa PAO1 based on the available crystal structure of the homologous protein in E. coli (PDB number 5IPL, chain F). Homology modeling was performed with I-TASSER (68). The N terminus is colored in dark blue, and the C terminus is colored in red. The seven different mutant residues (Table 2) are indicated in white. Fs denotes a frameshift, and * denotes a stop codon.

The P. aeruginosa *rpoS* gene encodes an alternative sigma factor of 334 amino acids in length. The various nonsynonymous *rpoS* mutations result in an amino acid substitution (*rpoS1*, *rpoS3*, and *rpoS4*), a truncation (*rpoS2*), a frameshift (*rpoS5* and *rpoS6*), or a lost start codon (*rpoS7*) (Table 2). We also mapped each RpoS mutation onto a three-dimensional protein structure (Fig. 1), indicating no particular domain preference or clustering. *rpoS1*, *rpoS2*, and *rpoS4* harbor mutations in a DNA-binding region that presumably disrupt promoter recognition and transcription initiation (29). *rpoS5*, *rpoS6*, and *rpoS7* encode either a nontranslated or an aberrant protein. As such, we presume that each mutation results in at least partial loss of function.

**TABLE 2:** RpoS mutations and associated NAG growth phenotypes

Strain/isolate^*a*^	Allele^*b*^	Change (position)^*c*^	NAG phenotype	
			Solid^*d*^	Liquid (OD_600_)^*e*^
PAO1 WT	*rpoS* (wild type)	None	+	1.3 ± 0.1^*f*^
PAO *rpoS5*	*rpoS5*	Frameshift (20)	−	0.059 ± 0.004
TR02	*rpoS1*	R308T	+	0.19 ± 0.01^*f*^
TR11	*rpoS2*	E295*	−	0.052 ± 0.001
TR12	*rpoS2*	E295*	−	0.055 ± 0.003
TR13	*rpoS3*	P210L	−	0.057 ± 0.004
TR16	*rpoS4*	G284S	−	0.089 ± 0.004
TR22	*rpoS5*	Frameshift (20)	−	0.062 ± 0.001
TR25	*rpoS6*	Frameshift (198)	−	0.057 ± 0.003
TR28	*rpoS7*	M1I	−	0.062 ± 0.012

a Evolved isolates TR02 to TR28 harbor mutations other than those in *rpoS*.

b Allele designation according to isolate number.

c Amino acid change relative to the RpoS protein sequence of strain PAO1. *, stop codon.

d +, NAG positive; −, no or marginal growth.

e Data represent means ± standard deviations (*n *= 3).

f *P* value of <0.05, indicating statistically significant difference from all other values.

To investigate the nature of the fitness costs and benefits conferred by the loss of *rpoS*, we chose *rpoS5* for further analysis. An early frameshift at codon 20 very likely results in a complete loss-of-function protein, thereby simplifying interpretations of mutant phenotypes (Table 2). We cloned the *rpoS5* allele and transferred it into the PAO1 WT to analyze the effects of this mutation. For comparison, we also utilized a previously constructed *rpoS* loss-of-function mutant in which the *rpoS* gene was disrupted by the insertion of a gentamicin resistance (Gm^r^) cassette (11).

### RpoS mutant frequency during *in vitro* evolution.

First, we considered the sequence of major mutational events during *in vitro* evolution. We know from previous work that *psdR* mutations rise to fixation by day 4 of subculturing and that *lasR* mutations comprise, on average, about a quarter of the population at day 12 (22). To evaluate how early *rpoS* mutations emerge, we sequenced the *rpoS* gene of 20 class I isolates from day 12 (23). None of the isolates harbored a mutation, indicating that *rpoS* mutations are selected gradually from within an established *psdR* mutant population.

**FIG 2:**
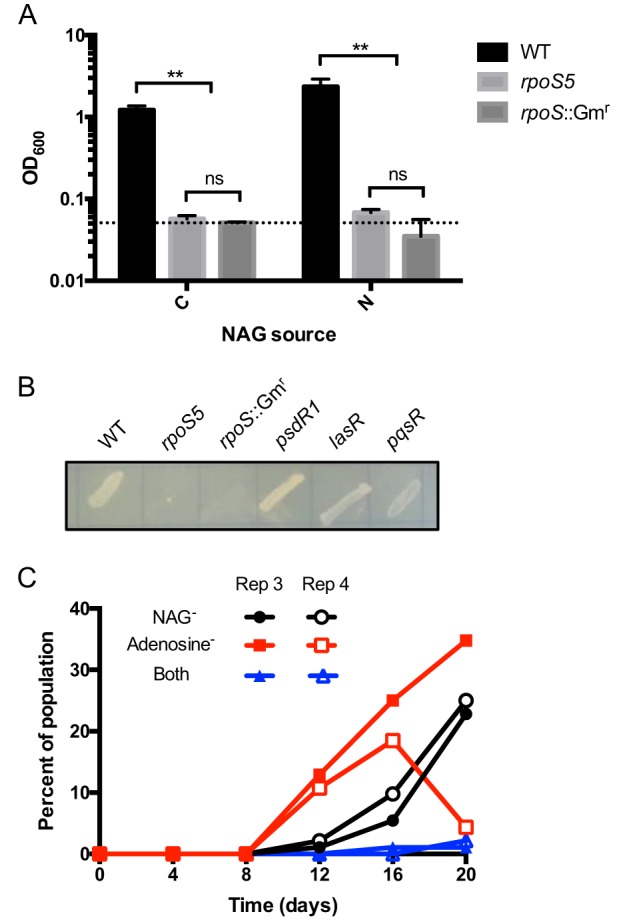
NAG assay and frequency of evolved mutations. (A) Growth of P. aeruginosa WT, *rpoS5*, and *rpoS*::Gm^r^ strains in liquid cultures containing M9 minimal medium with NAG as either sole C or sole N source. Culture density (OD_600_) was measured after 18 h of growth. The dashed line indicates the inoculation density of OD_600_ (0.05). Error bars indicate standard deviations of the means (*n *= 3). **, *P < *0.001; ns, not significant. (B) Growth of different P. aeruginosa strains on solid M9 minimal medium with NAG as the sole N source. The image was taken after 24 h of incubation. (C) Emergence of *rpoS* and *lasR*-deficient isolates during *in vitro* evolution in casein medium. “Rep 3” and “Rep 4” designate two independent biological replicates from Wilder et al. (23) and are consistent with the notation used in Table 1. Frequencies of isolates with no or marginal growth on NAG as N source, on adenosine as C source, or on both media are shown.

To more precisely quantify the frequency of *rpoS* mutants over time, we devised an assay suitable for large-scale screening of *rpoS*-deficient phenotypes. To this end, we used Biolog phenotype arrays to identify a specific growth condition that would enable differentiation of the WT strain from an *rpoS* mutant. Among 384 different C and nitrogen (N) sources, only one produced a strong phenotype (see Data Set S1 in the supplemental material). The WT strain grew on the compound N-acetyl-l-glutamic acid (NAG) as either a C or N source, whereas the *rpoS5* mutant did not. The same growth differences manifested in our M9-based minimal medium (Fig. 2A). The molecular basis of the NAG phenotype is not yet known. One pathway for NAG utilization appears to involve periplasmic conversion to glutamate by a deacetylase (30). However, the corresponding gene has not been identified and its identity is not apparent from our list of RpoS-induced genes (11).

10.1128/mBio.03090-19.2 DATA SET S1 Biolog screening. The assay plates used were PM1, PM2A, and PM3B. Growth of the P. aeruginosa WT strain and the defined *rpoS5* mutant is reported as OD_600_ in the first two columns. The color scheme distinguishes high growth (green), intermediate growth (yellow), and little to no growth (red). In the third column, the difference in OD_600_ between WT and *rpoS5* is reported, and the color scheme distinguishes differences in growth accordingly. Framed cells mark the growth conditions with the largest differences, i.e., NAG as a C source (position G3 on PM2A) and NAG as an N source (position D1 on PM3B). Download Data Set S1, XLSX file, 0.02 MB. Copyright © 2020 Robinson et al. 2020 Robinson et al. This content is distributed under the terms of the Creative Commons Attribution 4.0 International license.

Our screening assay for the identification of evolved *rpoS* mutant isolates was based on a solid minimal medium with NAG as the sole N source. The defined *rpoS5* and *rpoS*::Gm^r^ mutations conferred no growth on this medium, whereas all other major mutations previously described in our *in vitro* evolution system did (*lasR*, *psdR*, *pqsR*; Fig. 2B). We initially screened all of the sequenced isolates listed in Table 1 and qualitatively evaluated their growth on solid medium. We found a good correlation between the presence of an *rpoS* mutation and a growth defect on NAG. Of the eight sequenced isolates with an *rpoS* mutation, seven showed no to marginal growth (Table 2). Only one (isolate TR02 with *rpoS1*) showed WT-like growth. The resulting mutation—a single amino acid substitution near the C terminus of the protein—apparently has no major impact on RpoS function.

To obtain additional quantitative information about the degree of NAG deficiency, we grew all eight evolved mutants in NAG-liquid culture and determined cell densities (Table 2). In this format, all of the *rpoS* mutant isolates, including TR02, showed much reduced growth compared with the WT. However, consistent with the plate assay, TR02 grew to a significantly higher density than the other isolates. In contrast, all isolates with a WT *rpoS* allele showed robust growth on solid NAG medium that was indistinguishable from the level seen with the WT parent (Table 1). We conclude that our plate assay reliably identifies NAG-deficient isolates as *rpoS* mutants and is suitable for large-scale screening, while recognizing the limitation that a small fraction of partial loss-of-function *rpoS* mutants may escape detection.

We screened all available isolates from the Wilder et al. study (two replicate lineages, with 92 isolates each from days 4, 8, 12, 16, and 20). *rpoS* mutants were detected as early as day 12 and increased in frequency on days 16 and 20 (Fig. 2C). With seven to eight generations per 24-h cycle during experimental evolution (22), this increase in frequency amounts to an average growth rate of the *rpoS* mutant subpopulation that is 7% to 11% higher than that of the remaining population. Combining the *rpoS* data with the available information on *lasR*-deficient phenotypes (20, 23), we found that *rpoS* mutants lagged behind *lasR* mutants in frequency and that very few isolates were both *rpoS* and *lasR* deficient. Thus, mutations in *rpoS* arose largely independently of those in *lasR* and cooccurred in only two isolates (TR02 and TR16). In TR02, the *lasR* mutation appears to have emerged prior to *rpoS*, because another isolate from the same replicate experiment (TR09) was found to harbor the same *lasR* allele (A228V) with no *rpoS* mutation.

### RpoS mutants as social cheaters.

**FIG 3:**
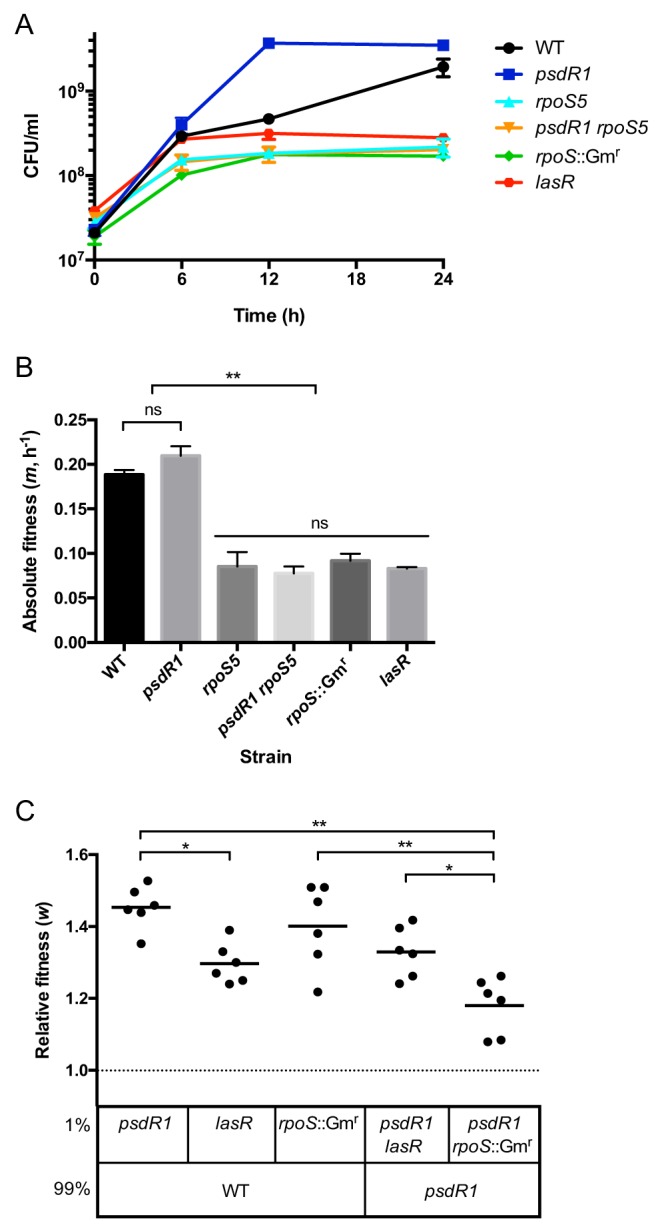
RpoS-dependent growth and fitness. The indicated P. aeruginosa strains were grown in casein medium for 24 h. (A) Growth in pure culture (measured in CFU per milliliter) at 0, 6, 12, and 24 h. Error bars indicate standard deviations of the means (*n *= 3). (B) Absolute fitness of strains shown in panel A. Absolute fitness was calculated as the average growth rate (Malthusian parameter) after 24 h. (C) Invasion of rare strains in coculture. Two strains were combined at 1:99 initial frequency, as indicated. Relative fitness values were calculated for the rare strain as the ratio of Malthusian parameters after 24 h. Means are plotted as horizontal lines, with individual replicates shown (*n *= 6). All means are significantly above 1 (*P < *0.001). *, *P < *0.05; **, *P < *0.001; ns, not significant.

To elucidate the phenotypic consequences of *rpoS* deficiency, we first evaluated the growth of individual strains in casein medium. We considered the effect of the *rpoS5* allele in the WT and in the *psdR1* mutant backgrounds. The *psdR1* mutation results in the deletion of six amino acids close to the carboxyl terminus conferring a complete loss of function (22). We compared the growth of the *rpoS5* mutant and the *rpoS5 psdR1* double mutant to that of the WT, the *psdR1* mutant, and the *lasR* deletion mutant (Fig. 3A). We also included the previously constructed *rpoS*::Gm^r^ insertion mutant (31). This strain grew identically to *rpoS5*, confirming the notion that the evolved *rpoS* allele confers complete loss of function. Whereas the *psdR1* mutant grew faster than the WT, the *rpoS5* mutant showed a substantial growth defect similar to that seen with the *lasR* mutant. Its highest density was reached within 6 h and plateaued between 6 and 24 h. Even the *psdR1 rpoS5* mutant followed this same pattern, indicating that a nonfunctional RpoS is detrimental to proteolytic growth in pure culture. Of note, initial low-density growth of the protease-deficient *lasR* mutant is routinely observed in this medium (20, 22, 23), presumably because of low-molecular-weight impurities in the commercial casein preparation. We also expressed the growth of all strains as absolute-fitness data (the average growth rate within a 24-h period; Fig. 3B) and assigned statistically significant differences to the patterns observed in Fig. 3A.

Even though *rpoS* mutants showed low absolute fitness, they were nevertheless prevalent in the population during experimental evolution (Fig. 2C), suggesting a high relative fitness in the presence of other *rpoS*-positive cells. We therefore tested the relative fitness of the *rpoS*::Gm^r^ mutant and the *psdR1 rpoS*::Gm^r^ mutant in coculture with either the WT or the *psdR1* mutant (Fig. 3C). Relative fitness is expressed as the ratio of average growth rates during the cultivation period. To distinguish strains in coculture, we employed *rpoS* mutant strains harboring the gentamicin resistance cassette for subsequent antibiotic selection. We were particularly interested in the ability of the mutants to invade a resident population when initially rare. Inoculated at 1% initial frequency, the *rpoS*::Gm^r^ and *psdR1 rpoS*::Gm^r^ mutants were enriched in WT and *psdR1* mutant cocultures, respectively. Likewise, the *lasR* and the *psdR1 lasR* mutants were enriched in their corresponding cocultures, confirming previous results (22). In all cases, the relative fitness of the rare strain is above 1. The relative fitness differences between strain pairs qualitatively match the sequence in which *psdR*, *lasR*, and *rpoS* mutations emerged during *in vitro* evolution (i.e., *w_psdR_*_versus WT_ > *w_psdR1 lasR_*_versus_ *_psdR_* > *w_psdR1 rpoS_*_versus_ *_psdR_*). In sum, we found that *rpoS* mutants have low absolute fitness in isolation but high relative fitness in mixed culture.

The combination of low absolute fitness with high relative fitness indicates that *rpoS* mutants, like *lasR* mutants, are social cheaters that take advantage of the secreted public goods supplied by others without investing in costly production themselves. This obligate, parasitic lifestyle depends on the presence of cooperators, consistent with the observation that *rpoS* mutants comprise only a subset of the evolved population (Table 1 and Fig. 2C).

### No apparent RpoS-dependent effect on proteolysis.

**FIG 4:**
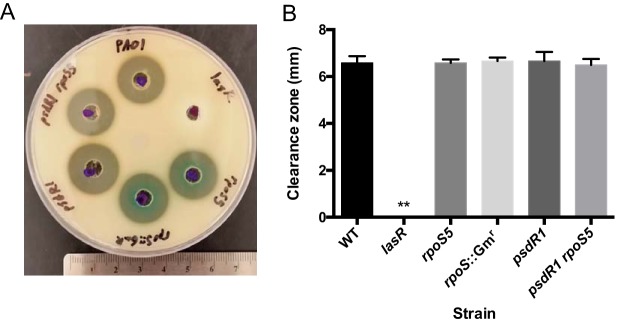
Skim milk proteolysis. The caseinolytic activity of different P. aeruginosa culture supernatants was quantified on skim milk agar plates. (A) Photograph of a representative plate. The corresponding strains are marked along the edge of the plate. The ruler on the bottom is in centimeters. (B) Quantitation of proteolytic activity. Zones of clearing were measured from the well edge to the end of the zone. Error bars indicate standard deviations of the means (*n *= 3). **, *P < *0.001 and significantly different from all other values.

Evidence thus far indicates that *rpoS* mutants are cheaters that may utilize public goods produced by the WT, most obviously secreted enzymes involved in casein proteolysis. We therefore tested the proteolytic capabilities of all our strains to identify differences (Fig. 4). To accomplish this, we measured the level of proteolysis on skim milk agar when incubated with cell-free culture supernatant. The supernatant was obtained from stationary-phase LB liquid cultures. These growth conditions promote protease expression but do not also require proteolysis for growth (22). All culture supernatants, with the exception of the *lasR* mutant supernatant, produced a clearance zone indistinguishable from that of the WT. Thus, proteolytic ability as assessed with our plate assay does not explain the behavior of the *rpoS* mutant.

We note that this assay is similar but not identical to the original skim milk plate assay used to classify evolved phenotypes (Table 1). The original skim milk assay qualitatively evaluates proteolytic activity through the zone of clearance surrounding a growing P. aeruginosa colony. The agar contains a small amount of LB medium, permitting some growth even of protease-deficient isolates. The supernatant assay shown in Fig. 4 is more quantitative because it strictly separates growth from proteolysis.

### Functions responsible for the RpoS phenotype.

To obtain additional insights into the mechanism underlying the *rpoS* cheater phenotype, we interrogated our previous microarray data of RpoS-dependent genes in P. aeruginosa PAO1 (11). Even though the RpoS regulon contains almost 800 genes, the information thus far obtained from our phenotypic characterization allows us to narrow our search. It is reasonable to assume that the cheater phenotype involves an RpoS-controlled public good, perhaps in the form of a secreted protease that escapes detection by the skim milk plate assay. This assay mainly detects the activity of the major casein endoprotease LasB elastase (32–34), but there are other extracellular proteases that might contribute to growth of P. aeruginosa in casein medium. Among the proteases secreted by P. aeruginosa, two are activated by RpoS (Table 3) (11). These are the aminopeptidase PaAP, encoded by *pepB*, and the endoprotease protease IV, encoded by *piv* (25, 35). PaAP seems the more likely candidate. Its high level of induction by RpoS correlates with the strong casein growth phenotype. PaAP preferentially cleaves leucine but also cleaves other amino acids from the ends of peptides (25). This activity would not be required for caseinolytic activity visualized on skim milk plates but might still promote growth on casein by making individual amino acids available from the breakdown products of other proteases.

**TABLE 3:** Extracellular proteases of P. aeruginosa and their regulation by RpoS

Gene no.	Gene name and product description	RpoS regulation^*a*^	Reference
PA0423	*pasP*, small protease	None	69
PA1249	*aprA*, alkaline metalloproteinase precursor	−1.7	70
PA1871	*lasA*, LasA protease precursor	None	71
PA2939	*pepB*, aminopeptidase PaAP	140	25
PA3724	*lasB*, LasB elastase, neutral metalloproteinase	None	71
PA4175	*piv* (*prpL*), protease IV	6.8	35
PA4541	*lepA*, large extracellular protease	None	72

a Fold change in gene expression of WT strain versus *rpoS* mutant (11). Downregulation is indicated with a minus sign.

**FIG 5:**
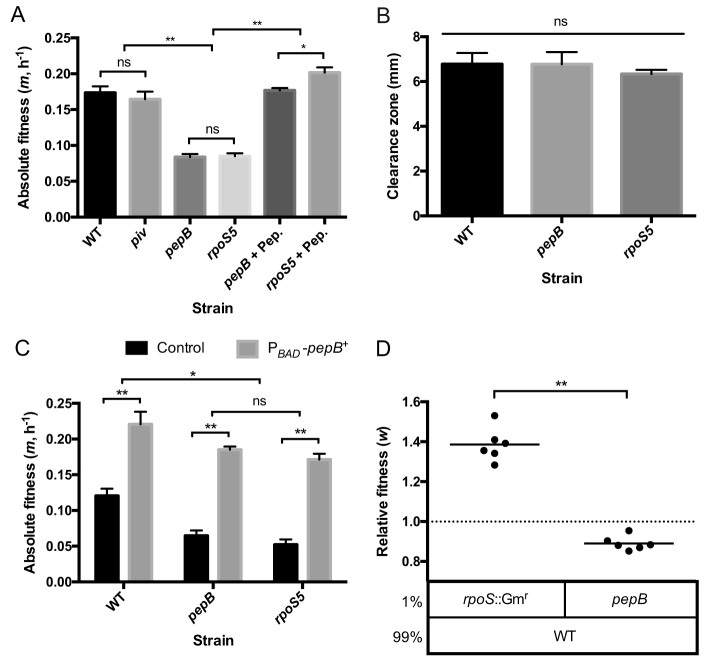
Fitness and proteolysis of *pepB* and *piv* mutants. (A) Absolute fitness (Malthusian parameter) of individual P. aeruginosa strains in casein medium after 24 h. Error bars indicate standard deviations of the means (*n *= 3). Leucine aminopeptidase was added to two of the cultures at time zero (+ Pep.). (B) Proteolytic activity of culture supernatants determined on skim milk plates. Zones of clearing were measured from the well edge to the end of the zone. Error bars indicate standard deviations of the means (*n *= 3). (C) Genetic complementation of individual strains in casein medium. Strains carried either a plasmid containing the arabinose-inducible *pepB* expression construct or a control plasmid. Absolute fitness (Malthusian parameter) was determined after 24 h. (D) Relative fitness levels in casein coculture. Each of the two mutant strains was combined with the WT at 1:99 initial frequency as indicated. Relative fitness values were calculated for the mutant strain as the ratio of Malthusian parameters after 24 h. Means are plotted as horizontal lines, with individual replicates shown (*n *= 6). Both means are significantly different from 1 (*P < *0.001). *, *P < *0.05; **, *P < *0.001; ns, not significant.

In order to examine the roles of PaAP and protease IV in proteolytic growth, we constructed *pepB* and *piv* mutants. The *piv* mutant grew as well in casein medium as the WT, ruling out its involvement in the *rpoS* mutant phenotype (Fig. 5A). The *pepB* mutant, in contrast, showed impaired growth in casein medium but showed no deficit in casein proteolysis on skim milk plates (Fig. 5A and B). These phenotypes are identical to those of the *rpoS* mutant. To confirm that the *pepB*-encoded enzyme, PaAP, is the only factor contributing to the loss-of-function growth phenotype of the *rpoS* mutant, we conducted two different complementation assays. First, we added purified leucine aminopeptidase to our casein minimal medium and found that growth was restored in both the *pepB* and *rpoS* mutant strains (Fig. 5A). Second, we ectopically expressed *pepB* from an arabinose-inducible promoter and found that it fully complemented the growth defect of the *rpoS* and *pepB* mutants (Fig. 5C). The absolute fitness of all three genetically complemented strains was above that of the noncomplemented WT, presumably because *pepB* is produced at high levels as the result of immediate induction upon inoculation.

Next, we investigated relative fitness in WT cocultures. The *pepB* mutant had a relative fitness level significantly below that of the *rpoS* mutant (Fig. 5D), consistent with the idea that the cost savings accruing from loss of an individual cooperative gene is lower than the savings associated with loss of an entire regulon (36). The relative fitness level of the *pepB* mutant was even slightly below 1, as the *pepB* mutant grew less well than the WT in coculture. However, this value is above what would be expected if the *pepB* mutant did not benefit at all from the WT (0.89 ± 0.04 versus 0.55 ± 0.03, *P* value < 0.001 by two-sample *t* test; see Materials and Methods for details), suggesting that PaAP is indeed secreted and at least partially beneficial to nonproducing cells.

**FIG 6:**
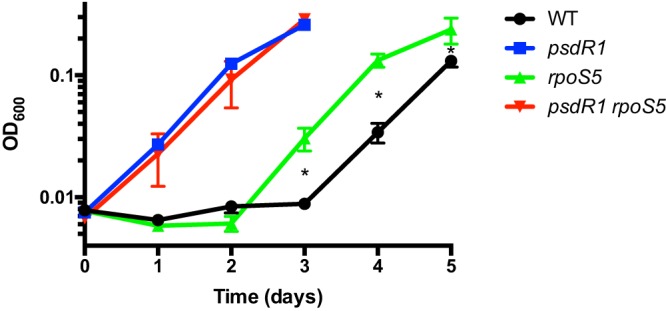
Dipeptide utilization. The indicated P. aeruginosa strains were grown in M9 minimal medium with Gly-Glu as the sole C source. Cell densities (OD_600_) were measured daily. Error bars indicate standard deviations of the means (*n *= 3). Among the comparisons between the WT and *rpoS* strains, the values representing days 3, 4, and 5 are significantly different (*, *P < *0.05). Among the comparisons between the *psdR1* and *psdR1 rpoS5* strains, none of the values at each time point are significantly different.

Considering the large size of the RpoS regulon, it is conceivable that some functions, particularly those repressed by RpoS, also provide a direct fitness benefit to the mutant during proteolytic growth. Interrogation of our previous transcriptome analysis identifies one candidate function, amino acid and dipeptide uptake (encoded by the *mdp*/*dpp* gene cluster) (11). The level of expression of these genes is about 2-fold-to-3-fold higher in the *rpoS* mutant than in the WT, providing a potential additional growth advantage to the cheater in mixed culture. Intriguingly, the same function is derepressed in *psdR* mutants, with about 50-fold-higher expression of *mdpA* and *dppA* in the mutants than in the WT (22). Given that *psdR* mutations arise before *rpoS* mutations during experimental evolution, it is questionable, however, whether the modest regulation by RpoS would have any additional effect. We tested these predictions with a dipeptide growth assay that measures the ability of strains to utilize the dipeptide Gly-Glu as the sole C source (22, 24). As reported previously (22, 24), the *psdR* mutant had a significant growth advantage compared with the WT (Fig. 6). The *rpoS* mutant also had a slight growth advantage compared with the WT, but the *rpoS psdR* mutant had no advantage compared with the *psdR* mutant, confirming our predictions. Because all evolved *rpoS* mutants harbor *psdR* mutations, the small effect of RpoS on *mdp*/*dpp* expression does not appear to contribute to their enrichment.

### A model that reconciles relative fitness differences between *rpoS* and *pepB* mutants.

The RpoS control of PaAP explains why *rpoS* mutants grow well in WT coculture but not in isolation. What still needs some explaining is the difference in relative fitness between *rpoS* and *pepB* mutants. As indicated above, there likely is a substantial metabolic burden associated with expressing RpoS-dependent genes. Most genes are induced by RpoS, and the average level of induction is much higher than the average level of repression (11). This metabolic burden is relieved by loss of RpoS, but not by loss of PaAP alone, translating to lower fitness costs and a higher growth rate. However, the question remains why the relative fitness of the *pepB* mutant is even lower than that of the WT. A plausible explanation is that PaAP-producing cells have preferential access to the secreted enzyme, even in a well-mixed environment. For example, if PaAP were not fully secreted and remained partially associated with the cell envelope, then digestion products might be directly captured by the producer before diffusing away. This property has indeed been reported for some microbial public goods (37, 38).

**FIG 7:**
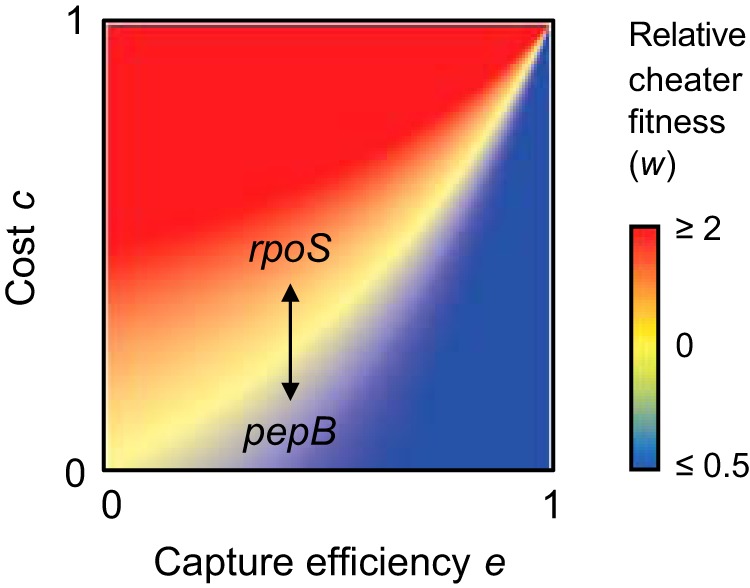
Mathematical model of relative fitness as a function of fitness costs and capture efficiency. Relative cheater fitness is expressed as the ratio of growth rates (Malthusian parameters) of the cheater and the WT cooperator. Red areas indicate higher cheater fitness, blue areas indicate higher cooperator fitness. Fitness costs and capture efficiency of the cooperator range between 0 (lowest) and 1 (highest). The approximate positions of the *rpoS* and *pepB* cheater mutants, resulting from a difference in the respective cost savings in gene expression, are indicated.

We conceptualized the interplay of the two factors, fitness costs on the one hand and capture efficiency on the other hand, in a simple mathematical model. We modeled the relative fitness of the cheater as the ratio of the cheater growth rate to the cooperator growth rate, the same metric as that used in our coculture assays. We expressed growth rates as a saturation function that is dependent on the concentration of the digestion products of PaAP as the relevant public good. Costs for the production of PaAP or for other pleiotropically linked traits reduce the growth rate of the cooperator, and capture efficiency reduces the growth rate of the cheater. As expected, the model shows that the cooperator does better when fitness costs are low and capture efficiency is high (Fig. 7). Conversely, the cheater does better when costs are high and capture efficiency is low. The difference in cost savings can explain a relative fitness level above 1 for the *rpoS* mutant and a relative fitness level below 1 for the *pepB* mutant.

## DISCUSSION

In this study, we identified a social phenotype in the RpoS response of the opportunistic pathogen P. aeruginosa. RpoS was originally described for E. coli as a master regulator of a stress response to nutrient exhaustion in stationary phase (39). In P. aeruginosa, RpoS also controls a large number of genes in stationary phase, although its role in stress protection is less clear (11–13). We used a variety of methods to investigate *rpoS* mutations and the nature of their fitness benefit during proteolytic growth of P. aeruginosa, including a novel screening assay based on NAG as a strictly RpoS-dependent nutrient. In our *in vitro* evolution experiments, we detected exclusively nonsynonymous loss-of-function mutations in *rpoS* that continuously increased in frequency, indicating a positive selection process (Table 1 and Fig. 2C). We provided evidence that the selected phenotype is social and exhibits the hallmarks of cheating behavior: RpoS mutants had low absolute fitness in pure culture but high relative fitness in coculture with cooperating cells (Fig. 3). Cheating has been observed in several microbial systems, including QS, nutrient acquisition, and development (19, 20, 40–42). It is considered a central problem in the evolution and maintenance of cooperative behavior, although mechanisms of cheater control have been described (21, 43).

The social phenotype of *rpoS* mutants can be explained by two properties: (i) the regulatory effect of RpoS on a single gene, *pepB*, that encodes the aminopeptidase PaAP (Fig. 5) and (ii) the large fitness cost associated with expressing genes in the global RpoS regulon (Fig. 3 and 7). Regarding the first point, we found that PaAP is necessary for growth on protein as nutrient source and that its presence is sufficient to complement the growth defect of an *rpoS* mutant (Fig. 5). PaAP has been characterized as a Zn-dependent metallopeptidase that preferentially cleaves leucine residues from the N termini of peptides (25). *In vitro*, it has at least 10-fold-lower activity toward other residues (25). However, given the strong phenotype, we surmise that hydrolysis of residues other than leucine likely contributes to proteolytic growth *in vivo*. PaAP is a highly processed extracellular protease secreted by the Esx type II secretion pathway (44). It is found in soluble form in the extracellular milieu and as an abundant component of outer membrane vesicles that can affect the formation of biofilms (27, 45). Consistent with its extracellular localization, we found that PaAP functions as a public good that is shared between WT and *rpoS* mutant cells.

Because the fitness of a targeted *pepB* mutant is lower than that of the WT in coculture, we propose that PaAP-producing cells have preferential access to the secreted enzyme or its products, even in a well-mixed environment. It is possible that PaAP is not fully secreted and remains partially associated with the cell envelope, such that digestion products are preferentially available to the producer. This property has been reported for the enzyme invertase in yeast (37) and has been suggested for the siderophore enterochelin in E. coli (38). The presumed localization of PaAP on the surface of P. aeruginosa outer membrane vesicles indeed renders the idea of a transient association with the cell envelope during the process of vesicle biogenesis plausible (46, 47).

The second point explains the large relative fitness benefit associated with the *rpoS* mutant. While PaAP represents an individual cooperative trait, RpoS is a pleiotropic regulator that controls many other traits (11). There is a substantial metabolic burden associated with expression of hundreds of RpoS-dependent genes, and this burden is relieved by loss of RpoS but not by loss of PaAP alone. The same reasoning explains why mutations in another pleiotropic regulator, LasR, confer a large fitness benefit, whereas mutations in an individual LasR-controlled public good, elastase, do not (36). Consistent with this argument, *in vitro* evolution did not select for mutations in *pepB* or other protease genes. In turn, there is no pleiotropic cost associated with expression of other genes in the RpoS regulon. Under the specific conditions of cooperative growth, all but one of the genes controlled by RpoS are indeed dispensable. Instead, we provide evidence for a potentially beneficial function, peptide uptake and metabolism, that is enhanced in the absence of RpoS and that could add to the fitness advantage acquired from cheating. RpoS mutations are not reported for P. aeruginosa isolates from infections and natural environments, suggesting that the associated growth conditions are more varied, favoring a functional RpoS response in these contexts.

When we consider *rpoS* in the context of the other major mutations in our evolved isolates, the genotype-phenotype relationship is not always clear (Table 1). For example, we do not know why isolate TR02 with *psdR*, *lasR*, and *rpoS* mutations represents a class I phenotype whereas isolate TR09 with identical *psdR* and *lasR* mutations represents a class III phenotype. There may be as-yet-unknown interactions between the mutations, and there may be additional mutations that we did not identify, due to either sequencing gaps or limited sample size. What we can say is that the *lasR* A228V allele in isolates TR02 and TR09 retains partial function (22), explaining why they are not categorized as fully QS-deficient class II isolates. Of note, we did not identify mutations in the orphan QS regulator PqsR in any of our isolates. Our previous study revealed fluctuating frequencies of *pqsR* mutations that were as high as 60% on day 12 and as low as 6% on day 20 (23), suggesting that we may have missed *pqsR* mutations from day 20 simply due to limited sampling depth. Limited sampling likely also explains why we detected no *rpoS* mutations in one of the four replicates.

The relative abundances of *psdR*, *lasR*, and *rpoS* mutations during *in vitro* evolution generally match their relative fitness differences in defined coculture (Fig. 2C and 3C) (22). Of course, there are additional factors that can influence mutant dynamics during *in vitro* evolution, such as differences in the abundances of preexisting mutations in the ancestral population, different propensities for loss-of-function mutations due to differing protein sizes and functions, and fitness contributions from other evolved mutations. We also found that the two major cheater lineages, *rpoS* and *lasR*, evolved largely independently of one another (Fig. 2C). This finding is consistent with the large overlap of the RpoS and LasR regulons (11), such that a second mutation would provide only a small additional reduction in fitness costs.

Our work highlights the power of experimental evolution, which complements targeted genetic analysis in the identification of functions in novel contexts. The literature on stress responses tends to emphasize nonsocial functions that protect the cell from harm. However, regulation of social functions makes sense if stress responses are interpreted as a response to ecological competition (5). Such a response is contingent on perception of nutrient limitation as interference competition from other microbes, and an adequate response to competition would be a counterattack through expression of cooperative secretions (e.g., toxins or antibiotics). While PaAP shares this cooperative property, there is no evidence that it also provides a competitive advantage by, for example, harming other cells or making digestion products available solely to P. aeruginosa. Beyond the microbial realm, recent research has shown complex, opposing effects of stress on cooperative behavior. Humans, for example, are inclined to cooperate under certain stressful situations, such as those requiring the immediate rescue of a person in danger (48). Female meerkats, in contrast, are less willing to cooperate with group members under conditions of stress imposed by dominant females (49).

Another important question relates to the general stability of cooperative populations during experimental evolution: can our comprehensive sequence analysis provide insights? We had previously observed that only one of five replicate populations collapsed after 20 days (approximately 200 generations) of experimental evolution, despite the rise of *lasR* cheater mutants (20, 22, 23). In this study, we identified yet another cheater type among the members of a phenotypic class previously considered to be “cooperators.” Both RpoS and LasR cheaters are deficient in proteolysis, because *pepB* expression requires both LasR and RpoS. Thus, the associated cheater phenotypes are not complementary in the sense that the two cheat with respect to different traits (50). Nevertheless, it is possible that one cheater might be able to restrain the other. RpoS mutants overproduce the redox-active metabolite pyocyanin, and pyocyanin preferentially inhibits *lasR* mutants (51). In addition, as mentioned above, pervasive mutations in *psdR* provide a demonstrated nonsocial fitness advantage that defers population collapse. Some of the other novel mutations that we identified primarily in class II (“hybrid”) isolates may work similarly. Class II isolates also harbor partial loss-of-function mutations in *lasR* that may “streamline” the QS regulon with respect to essential functions, thereby optimizing metabolic costs (22). Finally, the long-term trajectory of the *in vitro*-evolved populations beyond the 20-day growth period is not clear, and it is possible that they would all collapse eventually.

**FIG 8:**
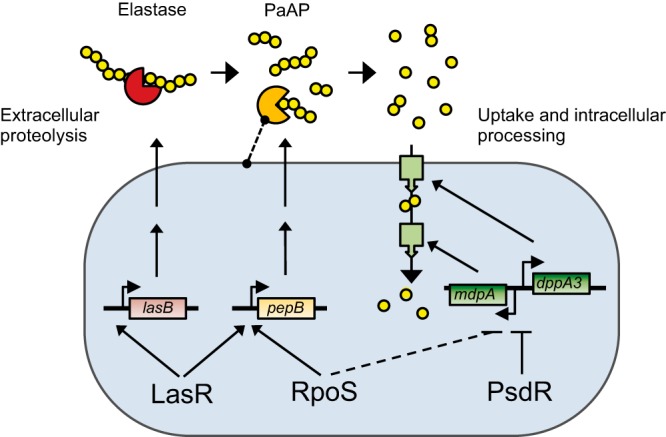
Schematic model of P. aeruginosa proteolytic growth. The model emphasizes regulatory pathways involved in nonsocial adaptation and cheating during experimental evolution. QS, via the regulator LasR, activates expression of the extracellular endoprotease elastase (brown; encoded by *lasB*) that breaks casein peptides into smaller fragments (yellow chains; each circle represents one amino acid). Both RpoS and LasR activate expression of the extracellular aminopeptidase PaAP (orange; encoded by *pepB*) that cleaves individual amino acids from the ends of peptides. A potential association of PaAP with the cell envelope is indicated. Loss of LasR or RpoS regulation prevents expression of the respective extracellular enzymes, producing a cheater phenotype that benefits from the enzymes produced by cooperator cells. PsdR represses genes (d*ppA3* and *mdpA*) involved in the transport and intracellular processing of amino acids and dipeptides (green). Loss of PsdR leads to increased uptake and processing, representing a nonsocial adaptation. RpoS has a slight inhibitory effect on uptake and processing genes (dashed line), potentially further contributing to a nonsocial fitness increase in an *rpoS* mutant.

As mentioned, the expression of *pepB* is controlled not only by RpoS but also by the QS regulator LasR (11). Thus, *pepB* regulation integrates two factors, the density of self-cells and stress. The signaling mechanisms in QS are well understood, but the cues that trigger the RpoS response in P. aeruginosa are not. Nutrient starvation is likely an important trigger, perceived through the stringent response that directly senses the lack of amino acids (52, 53). It is intriguing to speculate that the Lrp protein in P. aeruginosa, a homolog of the leucine-responsive protein in E. coli, is involved in sensing the products of leucine aminopeptidase and promoting RpoS-dependent transcription (54). In any case, it seems clear that the presence of a high density of self-cells ensures that peptidase secretion is effective, while nutrient stress provides information about the actual need for this enzyme. It is useful here to consider PaAP in the context of other proteases secreted by P. aeruginosa. Endoproteases and aminopeptidases likely work together during proteolytic growth (Fig. 8). Endoproteases such as LasB elastase break down peptide strands into smaller fragments (55, 56). Elastase is activated by QS but not by RpoS (11, 31). Thus, high cell density alone seems to be sufficient to anticipate a future need for this enzyme. PaAP is triggered by both cell density and starvation. PaAP frees individual amino acids from the peptide fragments that are taken up by the cell. Consistent with this scenario, proteolytic activation of PaAP seems to require elastase (25).

Taken together, we have provided evidence for a role of global stress responses beyond cellular protective functions. For the RpoS response in P. aeruginosa, one such role is the utilization of alternative nutrient sources available through cooperative secretions vulnerable to exploitation by cheaters.

## MATERIALS AND METHODS

### Strains and culture conditions.

For all experiments, we used the P. aeruginosa PAO1 wild-type (WT) strain or mutant derivatives. For a complete list of strains, see Table 4. Unless otherwise specified, strains were grown in Lennox lysogeny broth (LB; also referred to as Luria-Bertani medium) buffered with 50 mM 3-(*N*-morpholino)-propanesulfonic acid (LB-MOPS; pH 7.0). All cultures were incubated at 37°C with shaking at 250 rpm. Where indicated, antibiotics were used at the following concentrations: gentamicin (Gm), 50 μg/ml; tetracycline (Tc), 100 μg/ml; and trimethoprim (Tp), 100 μg/ml.

**TABLE 4:** Bacterial strains and plasmids

Strain or plasmid	Relevant property(ies)^*a*^	Reference or source
P. aeruginosa strains		
PAO1	Wild type	73
PAO Δ*lasR*	PAO1 derivative; Δ*lasR*; unmarked in-frame deletion from amino acids 102–216	23
PAO Δ*lasR*; Tp^r^	PAO Δ*lasR* derivative; chromosomal mini-Tn*7*–Tp^r^ insertion near *glmS* gene	23
PAO *rpoS5*	PAO1 derivative; *rpoS5*; unmarked mutant in which wild-type *rpoS* was replaced with evolved *rpoS5*	This study
PAO *rpoS*::Gm^r^	PAO1 derivative; *rpoS*::Gm^r^; marked mutant with *aacC1* inserted in the *rpoS* gene	11
PAO *psdR1*	PAO1 derivative; *psdR1*; unmarked mutant in which wild-type *psdR* was replaced with *psdR1*	22
PAO *psdR1*; Tc^r^	PAO *psdR1* derivative; chromosomal mini-Tn*7*–Tc^r^ insertion near *glmS* gene	22
PAO *psdR1* Δ*lasR*; Tc^r^	PAO Δ*lasR* derivative; unmarked double mutant harboring the *psdR1* and Δ*lasR* mutations; chromosomal mini-Tn*7*–Tc^r^ insertion near *glmS* gene	22
PAO *psdR1 rpoS5*	PAO *psdR1* derivative; unmarked double mutant harboring the *psdR1* and *rpoS5* mutations	This study
PAO *psdR1 rpoS*::Gm^r^	PAO *psdR1* derivative; marked double mutant containing both *psdR1* and *rpoS*::Gm^r^ mutations	This study
PAO Δ*pepB*	PAO1 derivative; Δ*pepB*; unmarked in-frame deletion from amino acids 148–376	This study
PAO Δ*pepB*; Tp^r^	PAO Δ*pepB* derivative; chromosomal mini-Tn*7*–Tp^r^ insertion near *glmS* gene	This study
E. coli strains		
DH5α	F^−^ Φ80*lacZYA-argF U169 recA1 hsdR17* (r_K_^−^ m_K_^+^) *phoA supE44* λ^−^ *thi-1 gyrA96* *relA1*	Invitrogen
SM10	*thi thr leu tonA lacY supE recA*::RP4-2–Tc::Mu Km^r^ λ*pir*	74
Plasmids		
pEX18Tc	Conjugative suicide plasmid; Tc^r^	57
pEX18Tc *rpoS*5	pEX18Tc with frameshift *rpoS5* allele	This study
pEX18Gm	Conjugative suicide plasmid; Gm^r^	57
pJN105	*araC*-pBAD cassette cloned in pBBR1MCS; Gm^r^	65
pSP401	PA2939 (*pepB*) in pJN105; Gm^r^	33

a Km^r^, kanamycin resistance.

For whole-genome sequencing, isolates were chosen from *in vitro* evolution experiments according to QS-dependent phenotypes (20, 22, 23). The PAO *rpoS5* and PAO *psdR1 rpoS5* mutants were constructed using a two-step allelic exchange as described previously (57). The *rpoS* region was PCR amplified with the desired evolved frameshift mutation using primers 5′-NNNNNNAAGCTTAGGTCGTCGATCGCAACGGTTC-3′ and 5′-NNNNNNTCTAGACGTCACTCGACAGGCCATTCTTCTC-3′ with flanking HindIII and XbaI restriction sites (underlined), respectively. The PCR fragment was cloned into XbaI-digested and HindIII-digested pEX18Tc for use in allelic exchange (57). The resulting construct was introduced into either the PAO1 WT or the previously constructed PAO *psdR1* mutant by conjugation (57). The markerless Δ*pepB* mutant was constructed as follows. An in-frame deletion of residues 148 to 376 was generated by splicing by overlap extension PCR (SOE-PCR), using four primers. Fragment one was amplified with 5′-NNNNNNGAATTCGGCGGGAAGAATTTGGTGATG-3′ (EcoRI site underlined) and 5′-CGACAGGTAGGTGAAATCCTTC-3′. Fragment two was amplified with 5′-GAAGGATTTCACCTACCTGTCGGGCAACTTCATCTATGACGGC-3′ (primer 2 complement underlined) and 5′-NNNNNNAAGCTTCTTCAACCTGTCGCCCAATC-3′ (HindIII site underlined). The Δ*pepB* construct was then cloned into HindIII-digested and EcoRI-digested pEX18Gm for use in allelic exchange as described above. The PAO *psdR1 rpoS*::Gm^r^ and PAO *piv*::Tc^r^ mutants were constructed by transformation with chromosomal DNA as previously described (58). Briefly, 1.25 ml of recipient cells from an LB overnight culture was pelleted, washed, and resuspended in 100 μl of 300 mM sucrose solution. Cells were electroporated after addition of 500 ng of purified chromosomal DNA from either strain PAO *rpoS*::Gm^r^ or strain DH0001 (PAO *piv*::Tc^r^) (14, 33). The *piv* mutation was transferred from one PAO1 strain to another to ensure isogenic backgrounds. Transformants were selected on LB plates containing the appropriate antibiotic and were verified by PCR.

### DNA preparation and sequencing.

Chromosomal DNA was extracted from 30 isolates evolved *in vitro* and from the PAO1 parent strain using a Gentra Puregene yeast/bacteria kit from Qiagen. DNA was suspended in 100 μl double-distilled water (ddH_2_O). Whole-genome sequencing was done using an Illumina HiSeq 3000 platform in the Center for Genome Research and Bioinformatics (CGRB) at Oregon State University, with 150-bp paired-end reads. Between 8,665,795 and 15,100,212 reads were obtained per sample (median, 12,497,204). Low-quality reads and primer sequences were trimmed using Trimmomatic (version 0.36) (59). Adapter sequences and 10 bases in leading or trailing positions were cut off, and reads that had a Phred score below 20 in a 4-base sliding window or were shorter than 40 bases were discarded (59). Reads were aligned, and genomes were assembled and manipulated with Bowtie2 (version 2.3.2) and SAMtools (version 1.3) (60, 61). Our PAO1 parent strain reads were aligned to the online reference P. aeruginosa PAO1 from the *Pseudomonas* Genome Database (https://www.pseudomonas.com), and evolved isolate reads were aligned to our parent strain (59, 62). Mutations were called using a Bayesian statistical method, FreeBayes (version v0.9.21-15-g8a06a0b) (63). Assembled genomes and mutations were visualized using Geneious (64). Targeted Sanger sequencing of *rpoS* was also conducted on DNA from 20 isolates from day 12 at the CGRB using the following primers flanking the gene: 5′-GCTTGAGTCGAACTCATGCAAG-3′ and 5′-CGGCATTTATCTACTTAGGCTCA-3′.

### Absolute and relative fitness assays.

To assess the growth and absolute fitness of individual strains, 4 ml of an LB-MOPS preculture was inoculated with a single colony from a freshly streaked plate and incubated for 18 h. A 4-ml volume of M9 minimal medium containing 1% (wt/vol) sodium caseinate as the sole C source was inoculated with a preculture aliquot to an optical density at 600 nm (OD_600_) of 0.05 and incubated for 24 h. CFU counts per milliliter were determined by serial dilution and spot plating. For enzyme complementation, M9-casein cultures were supplemented with 0.05 U/ml of leucine aminopeptidase (from porcine kidney; Sigma-Aldrich CAS no. 9054-63-1). For genetic complementation, plasmid pSP401 harboring *pepB* under the control of the arabinose-inducible P*_BAD_* promoter (33) was introduced into the mutant and WT strains. Controls carried parent plasmid pJN105 (65). M9-casein cultures were supplemented with 0.2% (wt/vol) l-arabinose at the beginning of growth. Absolute fitness was calculated as average growth rate or Malthusian parameter (*m*), with *m* = ln(*N*_1_/*N*_0_)/*t*, where *N*_1_ is the final CFU count per milliliter after 24 h, *N*_0_ is the initial CFU count per milliliter at time zero, and *t* is the culturing time in hours (66).

For cocultures, strain mixtures were formulated from individual LB-MOPS precultures according to OD_600_ readings. To distinguish the strains in coculture, the rare strain carried a chromosomal antibiotic resistance marker (Gm^r^, Tc^r^, or Tp^r^) (Table 4). Tc^r^ and Tp^r^ markers each reside on a mini-Tn*7* insertion at a neutral chromosomal site (67). They have no measurable effect on growth rate (23). A 600-μl volume of M9-casein minimal medium was inoculated with the strain mixtures in a 96 deep-well block at a starting OD_600_ of 0.02. Cocultures were incubated for 24 h. At 0 and 24 h, CFU counts per milliliter and strain ratios were determined by serial dilution and spot plating on LB plates with and without the respective antibiotic. Relative fitness values were calculated as the ratio (*w*) of the Malthusian parameters of the two competing strains (22, 23) as follows: *w*_cheater_ = *m*_cheater_/*m*_cooperator_.

A hypothetical relative fitness value was calculated for the *pepB* mutant in WT coculture at 1% initial mutant frequency, under the assumption that there is no social interaction between the two strains. The values corresponding to the initial and final coculture densities (CFU counts per milliliter at 0 and 24 h) necessary for this calculation were determined as follows. The initial coculture densities of both strains were taken directly from the experiment in Fig. 5D. The final coculture densities were inferred from the respective single culture densities of the experiment in Fig. 5A. Specifically, the final coculture density of a *pepB* mutant that did not interact with the WT would be 1% of its final density in single culture, representing the low growth yield attainable with a proteolysis defect. The final coculture density of a WT strain that did not interact with the *pepB* mutant would essentially be as high as that in single culture, because the *pepB* mutant frequency remained negligible.

### Proteolysis and adenosine growth assays.

To confirm previously recorded QS-dependent phenotypes of each *in vitro*-evolved P. aeruginosa isolate, we conducted qualitative skim milk proteolysis and adenosine growth assays (20). A single freshly streaked colony from each isolate was patched onto skim milk plates (4% skim milk–quarter-strength LB) and adenosine agar plates (M9 minimal medium with 0.1% adenosine as the sole C source) and incubated at 37°C. After 18 h, skim milk proteolysis was evaluated as the zone of clearance surrounding each colony. After 48 h, growth was evaluated on adenosine plates. For both assays, the respective phenotypes were compared to those of a positive WT control and a negative *lasR* mutant control.

For a more quantitative assessment of casein proteolysis, we measured the proteolytic activity of culture supernatant on skim milk plates. The QS-controlled enzyme LasB elastase is the major casein protease in this assay (32, 33). A liquid culture with 4 ml of LB-MOPS was inoculated with a single freshly streaked colony and grown for 24 h at 37°C. Cultures were centrifuged, and supernatant liquid was filtered (using a 0.2-μm-pore-size filter). Wells cut out of skim milk agar plates with the back side of a Pasteur pipette were filled with 200 μl of each culture supernatant. Agar plates were incubated for 24 h at 37°C. Each circular zone of proteolysis was measured from the well edge to the end of the zone.

### Phenotype arrays and *rpoS* mutant screen.

For Biolog screening, 96-well microtiter plates PM1, PM2A, and PM3B were used (Biolog Inc., Hayward, CA). PM1 and PM2A contained C sources, and PM2B contained N sources. The manufacturer’s protocol for E. coli and other Gram-negative bacteria was followed. For inoculation, a suspension was made in inoculating fluid IF-0 from fresh LB plate cultures grown overnight. For PM3B, 20 mM sodium pyruvate was added as a C source. The starting OD_600_ was 0.06 (85% transmittance). Plates were incubated for 24 h without shaking, and OD_600_ was measured with a multifunction plate reader (Tecan M200).

To determine the NAG growth phenotype of defined and evolved strains, M9 minimal salts medium was formulated with NAG (0.5% [wt/vol]) either as the sole C or N source. When NAG was the C source, 0.1% (wt/vol) NH_4_Cl was used as the N source. When NAG was the N source, 0.5% (wt/vol) sodium pyruvate was used as the C source. For liquid cultures, the strains were inoculated from LB stationary-phase cultures to an OD_600_ of 0.05. Cultures were grown for 18 h, and then OD_600_ was measured. For plate cultures, strains were either patched individually with a toothpick or plated with a 96-well replicator from freshly grown LB source plates. Growth phenotypes were scored visually after 24 h of incubation.

### Dipeptide growth assay.

To evaluate the ability of each strain to grow with a dipeptide as the sole C source, growth assays were performed as described previously (22, 24). Strains were precultured in M9 minimal medium with 0.5% (wt/vol) glucose for 18 h, washed in 1× M9 salts, and inoculated at an OD_600_ of 0.02 in 4 ml of M9 minimal medium containing 10 mM Gly-Glu dipeptide as the sole C source. Cultures were incubated at 37°C for up to 5 days, with OD_600_ measurements taken every day.

### Mathematical model.

We modeled the relationship between cooperator and cheater strains by focusing on their growth rates, which directly correlate with the relative fitness values measured experimentally. A full mechanistic model based on ordinary differential equations would have many degrees of freedom due to unknown enzymatic and growth parameters. Assuming Monod kinetics, a generic growth rate constant is defined as μ = μ_max_ * P/(*K_s_* + *P*), where μ_max_ is the maximum growth rate, *K_s_* is the half-saturation constant, and *P* is the concentration of the growth substrate, which in our case is the product of the PaAP enzyme. We introduce the product capturing efficiency *e* and the relative cost *c* of *rpoS*- and *pepB*-dependent gene expression, respectively, as fractions between 0 and 1. Direct capture by the cooperator reduces the level of *P* available to the cheater by the factor (1 − *e*), and specific gene expression costs incurred by the cooperator reduce its growth rate by the factor (1 − *c*). The growth rates for cooperator and cheater then correspond to the following equations:

$$\mu_{\text{cooperator}}=\left(1–c\right)*\mu_{\text{max}}*P/\left(K_{s}+P\right)$$

$$\mu_{\text{cheater}}=\mu_{\text{max}}*\left(1-e\right)*P/\left[K_{s}+\left(1-e\right)*P\right]$$

For simplicity, we assume equal μ_max_ and *K_s_* values for the two strains. The growth rate ratio of cheater to cooperator, which is equivalent to the ratio of Malthusian growth parameters or the relative cheater fitness parameter *w*, is then calculated as follows:

$$w=\mu_{\text{cheater}}/\mu_{\text{cooperator}}=\left(1-e\right)*\left(K_{s}+P\right)/\left\{\left(1-c\right)*\left[K_{s}+\left(1-e\right)*P\right]\right\}$$

We determined *w* for all combinations of *c* and *e* at a *P*/*K_s_* ratio of 1 and visualized the data as a heat map using Morpheus (https://software.broadinstitute.org/morpheus). Of note, costs incurred by the cooperator are equivalent to costs saved by the cheater. While the former reduces the cooperator growth rate by the factor (1 − *c*), the latter increases the cheater growth rate by the factor [1/(1 − *c*)]. Both result in the same *w*.

### Statistical analysis.

Statistical analysis was performed using GraphPad Prism version 7 (GraphPad Software, La Jolla, CA). Ordinary one-way analysis of variance (ANOVA) was used for Table 2, Fig. 3B and C, 4B, and 5A and B. Ordinary two-way ANOVA was used for Fig. 2A and 5C. Two-way repeated-measures ANOVA was used for Fig. 6. For all ANOVA variants, either Tukey’s or Sidak’s multiple-comparison test was used for pairwise comparisons (generally indicated with horizontal brackets in the figures). In some figures, a single horizontal line is used to summarize results of multiple pairwise comparisons with identical statistical results. A two-sample, unpaired Student's *t* test with equal variance was used for Fig. 5D, and a one-sample Student's *t* test was used for Fig. 3C and 5D. In all cases, the significance threshold was α = 0.05.
