# Supplementary figures and images for: Cooperation and Cheating through a Secreted Aminopeptidase in the Pseudomonas aeruginosa RpoS Response
Source: mBio. 2020 Mar 17;11(2):e03090-19. doi: 10.1128/mBio.03090-19 (PMC7078477; doi:10.1128/mBio.03090-19)

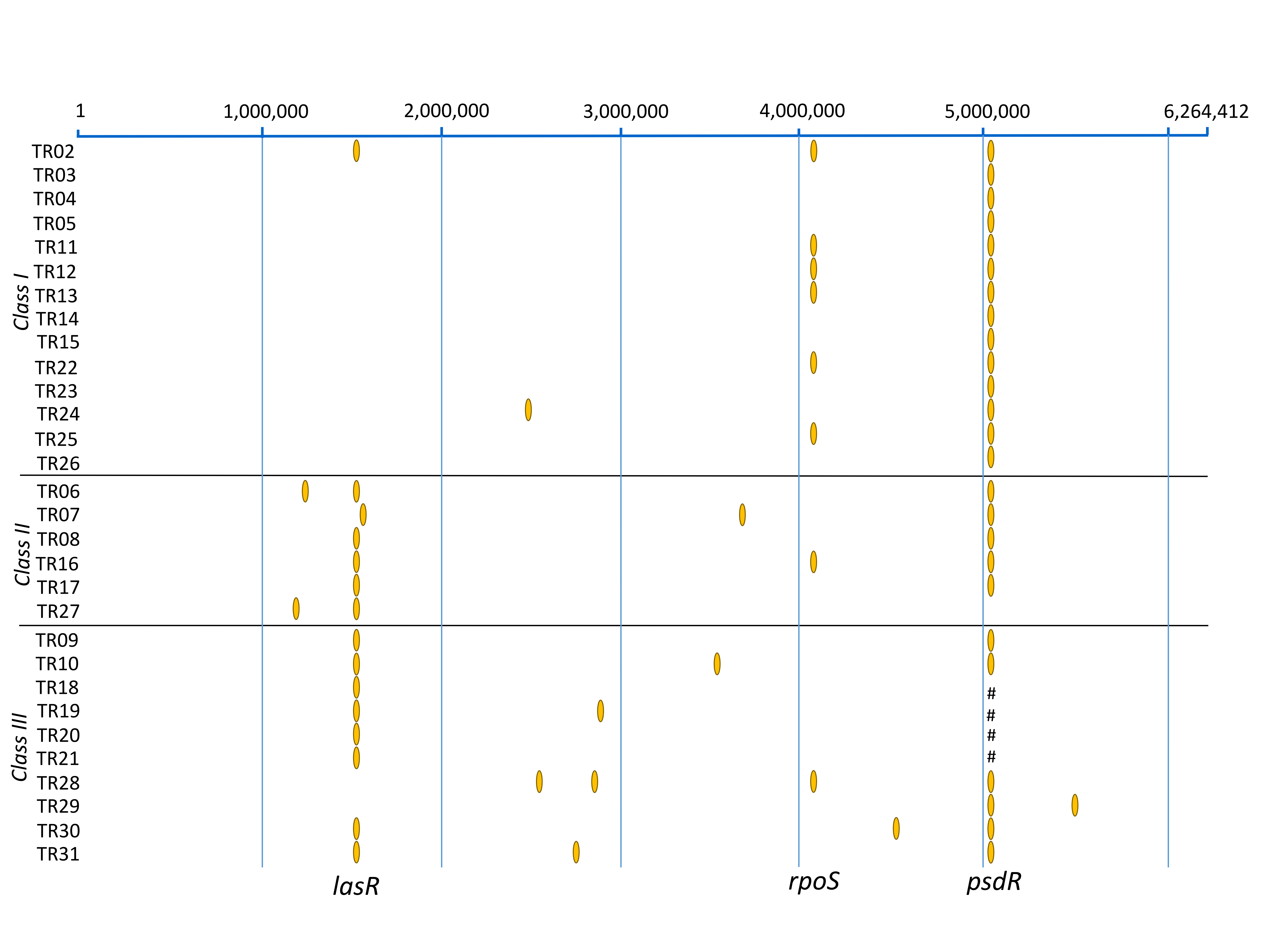

Supplement: FIG S1 [file mBio.03090-19-sf001.pdf]
